# The visibility paradox: algorithmic audience pressure, self-discrepancy, and psychological well-being in creative media professionals

**DOI:** 10.3389/fpsyg.2026.1986473

**Published:** 2026-10-08

**Authors:** Fangting Xu, Lizhao Wang

**Affiliations:** 1 Department of Art & Media Convergence, The Catholic University of Korea, Bucheon, Republic of Korea; 2 Department of Performing Arts, Graduate School of Digital Image and Contents, Dongguk University, Seoul, Republic of Korea

**Keywords:** algorithmic visibility, creative media professionals, creator well-being, media psychology, self-discrepancy, social evaluation

## Abstract

Algorithm-driven platforms increasingly mediate how creative media professionals reach audiences, build careers, and evaluate their own public visibility. Existing research has documented visibility labor, algorithmic precarity, creator stress, burnout, unwanted visibility, and traffic anxiety, yet the psychological process through which platform-mediated visibility becomes a persistent condition of self-evaluation remains less clearly theorized. This Perspective proposes algorithmic audience pressure as a conceptual lens for understanding that process. We define it as a persistent state of anticipated social evaluation in which creators expect their public self-presentations to be judged by audiences whose composition, scale, timing, and feedback are partly shaped by algorithmic distribution. Drawing on impression management and self-discrepancy theory, we argue that this pressure encourages chronic self-monitoring and can widen a creative–platform self-discrepancy between the creator one seeks to be and the platform-facing self one believes must be performed to remain visible. We formulate three testable propositions and describe a visibility paradox in which the same visibility that provides recognition, connection, and career opportunity can also undermine sustainable creative identity and psychological well-being.

## 1 Introduction

Algorithm-driven platforms have become more than channels through which creative work is distributed. For filmmakers, audiovisual creators, performers, independent producers, and digital storytellers, platforms increasingly shape discoverability, audience reach, reputation, monetization, and access to professional opportunities. Research on visibility labor and platform-dependent creative work shows that creators must continually make themselves and their work legible to platform systems while adapting to shifting expectations about what becomes visible (Abidin, 2016; Cotter, 2019; Duffy et al., 2021). This intermediation is intensified by platform infrastructures that rank, recommend, and circulate cultural content, encouraging creators to treat visibility as an ongoing occupational condition rather than a discrete outcome of publication (Hutchinson, 2023; Huang and Ye, 2024; Echauri, 2026).

The psychological consequences of this condition are increasingly visible. Studies on creators describe uncertainty about algorithmic distribution, continuous content demands, comparison with peers, blurred personal–professional boundaries, and exhaustion (Haug et al., 2024; Musgrave et al., 2025; Nguyen and Duffy, 2026). A recent systematic review similarly shows that algorithmic systems reorganize creator labor through visibility incentives, dependence, and unequal forms of control (Liang et al., 2025). Parallel work has begun to theorize traffic anxiety among digital laborers, emphasizing how traffic metrics can become objects of desire and distress (Fan, 2026). These findings make clear that creator well-being cannot be reduced to generic screen time or social-media use: for many creative workers, public visibility is embedded in the conditions of doing the job itself.

Yet two literatures remain only partially connected. Platform and cultural-labor research explains how creators pursue visibility under opaque and changing infrastructures, whereas psychological research explains how self-presentation, anticipated evaluation, and discrepancies between self-representations can affect emotion and well-being (Leary and Kowalski, 1990; Higgins, 1987; Bij de Vaate et al., 2020; Park et al., 2023). What remains less clearly theorized is the process by which algorithmically mediated visibility becomes a persistent social-evaluative environment. Algorithms do not merely alter the amount of exposure. They can change who encounters a creator, when exposure occurs, how responses are quantified, and how old content is recirculated, thereby reshaping the conditions under which an audience is anticipated (Bucher, 2017; Lee, 2025).

This Perspective develops three arguments. First, we propose algorithmic audience pressure as a conceptual lens for understanding how platform-mediated visibility becomes anticipated social evaluation rather than merely a distribution outcome. Second, drawing on impression management and self-discrepancy theory, we argue that such pressure can foster chronic self-monitoring and widen a creative–platform self-discrepancy between the creator one seeks to be and the platform-facing self one believes must be performed to remain visible. Third, we conceptualize visibility as paradoxical: it can generate recognition, connection, and opportunity while simultaneously creating psychological conditions that undermine sustainable creative identity. These arguments are formalized in three propositions that make the framework empirically testable and distinguish it from neighboring accounts of burnout, precarity, and traffic anxiety.

## 2 From algorithmic visibility to algorithmic audience pressure

Audience orientation is not new to creative work. What changes on algorithmic platforms is the instability of the audience itself. Social-media users already navigate imagined audiences and context collapse, because multiple social groups can converge around the same public content (Marwick and boyd, 2011; Davis and Jurgenson, 2014; Litt and Hargittai, 2016). Algorithmic distribution adds another layer: ranking and recommendation systems participate in deciding which publics encounter a creator and at what scale. Work on algorithmic imaginaries and algorithmic gossip shows that creators develop informal theories about platform systems and adapt their behavior accordingly, even when the operative rules remain opaque (Bucher, 2017; Bishop, 2019; Register et al., 2023). Platforms therefore shape not only visibility but also the creator’s expectations about visibility and recognizability (Gillespie, 2017).

This matters because creators do not perform before a stable or fully knowable public. Gallagher’s (2017) notion of algorithmic audiences captures how writers can orient themselves toward audiences produced partly through algorithmic processes. For contemporary creators, the composition, scale, and timing of the audience can be similarly contingent on recommendations, search systems, trending features, and recirculation. Visibility can also be unwanted. Lee (2025) shows how transnational Korean female creators experienced harmful algorithmic visibility when content reached racist, fetishizing, or otherwise unintended audiences, highlighting that the psychological meaning of visibility depends on audience context rather than exposure volume alone.

We therefore use the term algorithmic audience pressure to describe a persistent state of anticipated social evaluation in which creators expect their public self-presentations to be judged by audiences whose composition, scale, timing, and feedback are partly shaped by algorithmic distribution. We distinguish this focal psychological condition from its antecedents and consequences. Three structural characteristics of algorithmically mediated visibility are especially important as antecedents. First, unpredictability concerns uncertainty about when, where, and to whom content will be shown. Second, quantification makes audience response continuously legible through views, likes, shares, comments, follower counts, watch time, and related indicators. Third, persistence reflects both the need to renew visibility and the continuing searchability or recirculation of past content. In combination, these features shift audience evaluation from an episodic post-performance event toward a condition that can be anticipated before production begins (Haug et al., 2024; Register et al., 2023). Thus, unpredictability, quantification, and persistence characterize the visibility environment, whereas chronic self-monitoring, creative–platform self-discrepancy, and psychological exhaustion are proposed downstream responses.

Algorithmic audience pressure overlaps with, but is not identical to, neighboring concepts. Visibility labor concerns the work required to become and remain visible; algorithmic precarity concerns dependence on opaque and changeable platform systems; social-evaluative threat concerns actual, imagined, or possible negative evaluation; and traffic anxiety centers anxiety around traffic and viewership metrics (Abidin, 2016; Duffy, 2020; Park et al., 2023; Fan, 2026). Algorithmic audience pressure is narrower than general platform precarity but broader than anxiety about traffic numbers: it identifies anticipated judgment from human audiences whose access is partly assembled by algorithmic systems. Importantly, it can arise before a metric has risen or fallen, because creators may modify content and self-presentation in anticipation of who might see them and how that audience may respond. Table 1 summarizes these distinctions.

**Table 1:** Conceptual distinctions around algorithmic audience pressure.

Concept	Primary object	Typical focus	Why AAP is distinct
Visibility labor	Labor performed to gain and maintain attention	Effort and strategy of becoming visible	AAP concerns anticipated audience judgment once exposure is algorithmically allocated; it is a psychological condition rather than a labor practice.
Algorithmic precarity	Occupational dependence on opaque and changing platform systems	Instability, control, and insecurity	AAP is narrower: it specifies the evaluative pathway through which algorithmic exposure can become psychologically salient.
Social-evaluative threat	Actual, imagined, or possible negative evaluation	Threat to the self from social judgment	AAP contextualizes evaluation within algorithmically assembled audiences, quantified feedback, and persistent exposure.
Traffic anxiety	Anxiety centered on traffic, viewership, and metric pursuit	Psychological investment in traffic numbers	AAP can arise before traffic outcomes are known and includes who may evaluate the creator, not only how much traffic is obtained.
Algorithmic audience pressure	Persistent anticipated social evaluation under algorithmically mediated visibility	Anticipated judgment from algorithmically assembled audiences	Focal psychological condition, distinct from antecedent visibility characteristics and downstream self-monitoring, self-discrepancy, and well-being.

AAP, algorithmic audience pressure. The distinctions synthesize Abidin (2016), Duffy (2020), Park et al. (2023), and Fan (2026).

*Proposition 1. Algorithmically mediated visibility characterized by greater unpredictability, quantification, and persistence will be associated with stronger algorithmic audience pressure. This relationship will be stronger when platform-visibility dependence is high and audience-context fit is low.*

## 3 From audience pressure to creative–platform self-discrepancy

Impression-management theory provides a useful starting point for explaining why algorithmic audience pressure should matter psychologically. Leary and Kowalski (1990) distinguish between impression motivation—the desire to control how one is perceived—and impression construction—the process of deciding what impression to convey. Impression motivation becomes stronger when others’ evaluations are relevant to valued outcomes. For creative media professionals, audience impressions can be directly tied to followers, engagement, reputation, commercial opportunities, commissions, and future employment. Self-presentation is therefore not merely recreational. Research on entrepreneurial femininity, micro-celebrity, and influencer careers shows how visibility and professional identity become intertwined with the strategic presentation of the self (Duffy and Hund, 2015; Khamis et al., 2017; Hou, 2019).

Digital platforms also make self-presentation unusually inspectable. Public profiles operate partly as persistent exhibitions rather than only as momentary performances (Hogan, 2010). Selective self-presentation can be strategically edited in computer-mediated environments, but the same affordances can encourage repeated reflection on how the self appears to others (Walther, 2007). Reviews of online self-presentation further show that its well-being implications depend on what kind of self is presented and how that presentation is experienced (Bij de Vaate et al., 2020), while authentic self-expression has been associated with greater subjective well-being (Bailey et al., 2020). More broadly, the effects of social-network-site use on well-being depend strongly on the processes that use activates rather than exposure alone (Verduyn et al., 2017). For professional creators, continuously available performance metrics and feedback can therefore support chronic self-monitoring: repeated checking and adjustment of how one’s work and public persona are being received.

Self-discrepancy theory helps explain why this monitoring can become psychologically costly. Higgins (1987) argues that discrepancies among self-representations are associated with distinct forms of emotional vulnerability. Translating this logic to platform-dependent creative work, we distinguish between a creative self and a platform-ought self. The creative self concerns the creator one understands oneself as or aspires to become through craft, aesthetic judgment, professional values, and meaningful expression. The platform-ought self concerns the public-facing version of oneself that one believes must be performed to remain discoverable, legible, and engaging within a particular platform environment. This is not a claim that algorithms literally prescribe a personality. Rather, creators infer platform expectations from analytics, peer observation, platform advice, prior performance, and informal theories about algorithmic systems (Bucher, 2017; Bishop, 2019).

The resulting tension can be especially salient in audiovisual and performance work because the creator’s professional product and public identity often intersect. A filmmaker may value slower, aesthetically complex storytelling yet believe that platform visibility requires shorter hooks, frequent updates, recognizable formats, or a stable personal persona. A performer or digital storyteller may similarly adapt tone, genre, pacing, or self-disclosure to what they imagine a platform and its audiences will reward. Research on platform-specific self-branding shows that creative workers already re-fashion their digital personae according to imagined platform affordances and audience assumptions (Duffy et al., 2017), while algorithmic gossip documents how creators modify content in pursuit of visibility (Bishop, 2019). We conceptualize the perceived distance between the creator one seeks to be and the platform-facing self one feels required to perform as creative–platform self-discrepancy.

The proposed mechanism is therefore not simply that more posting produces burnout. Algorithmic audience pressure makes audience judgment chronically anticipatory; chronic self-monitoring increases attention to discrepancies between preferred and strategically performed selves; and sustained discrepancy can contribute to strain, dissatisfaction, and psychological exhaustion. Emerging creator evidence is consistent with parts of this account. Musgrave et al. (2025) document pressure to produce content, uncertainty, social comparison, identity negotiation, and exhaustion among musicians, while Nguyen and Duffy (2026) show that burnout has become a salient yet unevenly speakable problem in the creator economy. Fan’s (2026) traffic-anxiety framework further demonstrates that metrics can become psychologically charged. Our argument extends these observations by locating an identity-related mechanism in anticipated human evaluation rather than in workload or traffic metrics alone. Alternative online self-presentations can also feed back into self-understanding, underscoring the need to examine identity transformation rather than treating presentation as superficial display (Walther and Lew, 2022).

Creative media professionals are an analytically useful population for this framework because their work frequently combines cultural production, public presentation, and platform distribution. The category can include filmmakers, performers, independent audiovisual producers, digital storytellers, and other creators whose professional opportunities are increasingly entangled with platform visibility. We do not assume these occupations are psychologically identical. Rather, existing studies of influencers, creative workers, and multi-platform creators reveal a shared structural condition: creators must coordinate content, audience management, relational labor, and platform-specific presentation while retaining a recognizable professional identity (Abidin, 2016; Glatt, 2024; Ma et al., 2023). The strength and consequences of creative–platform self-discrepancy should therefore vary across occupations and platforms, making that variation an empirical question rather than a premise.

*Proposition 2. Algorithmic audience pressure will be associated with greater chronic self-monitoring and, through that monitoring, with greater creative–platform self-discrepancy.*

## 4 The visibility paradox

Visibility is not inherently harmful. For creators, platform exposure can provide recognition, audience connection, networking, self-expression, feedback, and access to opportunities that would otherwise be difficult to obtain. Musgrave et al. (2025), for example, describe both career-building and social benefits alongside the pressures experienced by musicians, and multi-platform creators actively prioritize platforms in ways that help them manage audiences and opportunities (Ma et al., 2023). Low visibility can therefore produce its own insecurity, particularly when professional survival depends on discoverability.

At the same time, high or unstable visibility can increase evaluative exposure. Metrics can make reception continuously comparable, opaque feedback can encourage repeated inference about what “worked,” and algorithmic recirculation can expose creators to audiences they did not intend to reach. These pressures may be unevenly distributed. Marginalized creators have reported self-censorship, unequal algorithmic visibility, and strategies for managing harmful or contextually inappropriate exposure (Duffy and Meisner, 2023; Lee, 2025). Relational labor can also intensify expectations that creators remain emotionally available and publicly authentic even when such availability is costly (Glatt, 2024).

We call this dual condition the visibility paradox: the same infrastructure that can sustain a creative career can also destabilize the psychological conditions needed to sustain creative identity. Visibility may generate opportunity through recognition and connection while simultaneously producing algorithmic audience pressure, chronic self-monitoring, creative–platform self-discrepancy, and exhaustion. These parallel pathways are summarized in Figure 1. The paradox implies that the relevant question is not whether visibility is intrinsically good or bad, but when visibility remains professionally useful without becoming psychologically intrusive.

**Figure 1:**
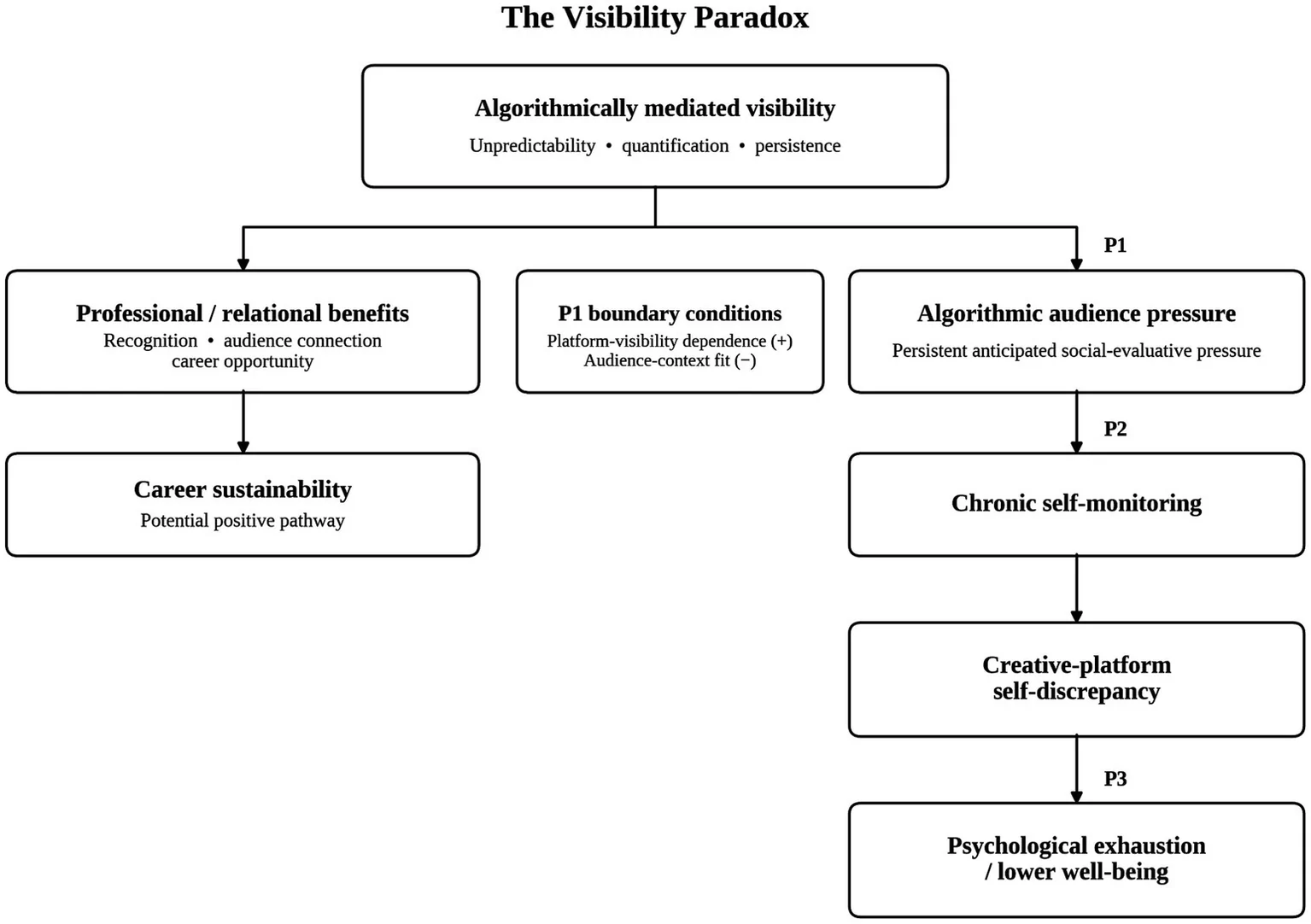
The visibility paradox model. Algorithmically mediated visibility can produce professional and relational benefits while also generating algorithmic audience pressure. Unpredictability, quantification, and persistence are treated as antecedent characteristics of the visibility environment rather than components of AAP. The proposed negative pathway proceeds through chronic self-monitoring and creative–platform self-discrepancy to psychological exhaustion and lower well-being. Solid arrows represent the proposed direct relationships. The box labeled P1 boundary conditions identifies the moderators of the relationship between algorithmically mediated visibility and AAP: platform-visibility dependence strengthens, whereas higher audience-context fit weakens this relationship. P1–P3 correspond to Propositions 1–3 in the text.

Two boundary conditions are expected to modify the relationship between algorithmically mediated visibility and algorithmic audience pressure. First, dependence on platform visibility should increase the goal relevance of audience evaluation: a creator whose income or access to work depends heavily on platform reach has less freedom to ignore volatile feedback. Thus, platform-visibility dependence should strengthen the extent to which unpredictable, quantified, and persistent visibility becomes anticipated social evaluation. Second, audience-context fit should shape whether exposure is experienced as supportive or threatening. Visibility to an intended professional community may strengthen recognition, whereas exposure to hostile, irrelevant, or identity-policing audiences may amplify pressure (Lee, 2025). Thus, higher audience-context fit should weaken this relationship, whereas lower fit should amplify it.

*Proposition 3. Creative–platform self-discrepancy will be associated with greater psychological exhaustion and lower well-being.*

## 5 Implications and future directions

The first priority is construct clarification. Future research should test whether algorithmic audience pressure is empirically distinguishable from technostress, social-media fatigue, algorithmic precarity, general social-evaluative threat, and traffic anxiety (Tarafdar et al., 2007; Bright et al., 2015; Duffy, 2020; Park et al., 2023; Fan, 2026). Qualitative work could first examine whether creators describe persistent anticipated audience evaluation and how it varies with the antecedent visibility conditions of unpredictability, quantification, and persistence. Subsequent scale development could then establish the convergent and discriminant validity of algorithmic audience pressure itself. Longitudinal and experience-sampling designs would be especially valuable because both visibility and evaluation fluctuate over time and because the proposed mechanism concerns repeated anticipation rather than one-time exposure.

A second priority is testing the identity pathway specified in the propositions. Research should examine whether algorithmic audience pressure predicts chronic self-monitoring, whether self-monitoring widens creative–platform self-discrepancy, and whether that discrepancy explains exhaustion or reduced well-being beyond workload and conventional platform stress. Cross-platform comparisons are important because platform architectures differ in recommendation systems, audience persistence, monetization, and identity norms. Multi-platform research already shows that creators prioritize, synchronize, and differentiate content across platforms (Ma et al., 2023). Comparisons among filmmakers, performers, musicians, digital storytellers, and influencers could clarify when public persona is central to the work and when platform adaptation is more easily separated from creative identity.

A third priority concerns inequality and design. Harmful visibility may be more consequential for creators whose identities or content attract harassment, stereotyping, or context collapse (Duffy and Meisner, 2023; Glatt, 2024; Lee, 2025). Platforms could therefore treat audience control as a well-being issue by improving tools for contextualizing distribution, limiting unwanted recirculation, filtering harassment, and making analytics less compulsively salient. Creative-media education should likewise move beyond production and distribution skills to include platform literacy, boundary management, psychological detachment, and strategies for maintaining continuity between creative values and public-facing work. These interventions should not place all responsibility on individual creators; the visibility environment is jointly produced by platform architecture, labor markets, audiences, and creator strategies.

## 6 Discussion

This Perspective argues that algorithmic visibility should be understood not only as a distributional or labor condition but also as a psychological environment of anticipated evaluation. By linking platform research with impression management and self-discrepancy theory, the framework explains why adaptation to algorithms may alter more than posting frequency or workload. Under uncertain, quantified, and persistent exposure—especially when recognition and opportunity depend strongly on platform visibility—creators may repeatedly monitor how they appear and gradually separate the self they wish to enact creatively from the self they believe a platform requires. The visibility paradox captures the resulting tension: visibility can be professionally enabling and psychologically demanding at the same time.

The framework is conceptual and does not establish causal effects. Algorithmic audience pressure has not yet been validated as a clinical or psychometric construct, and creator occupations, platforms, business models, and cultural contexts differ substantially. These limits are reasons for empirical testing rather than grounds for assuming a universal effect. Future work should therefore examine when the proposed pathway emerges, for whom it is strongest, and which platform or occupational conditions interrupt it. Understanding creator well-being ultimately requires asking not only whether creative work becomes visible but how, to whom, and under what psychological conditions that visibility is produced.
